# Osteoprotegerin Prevents Development of Abdominal Aortic Aneurysms

**DOI:** 10.1371/journal.pone.0147088

**Published:** 2016-01-19

**Authors:** Batmunkh Bumdelger, Hiroki Kokubo, Ryo Kamata, Masayuki Fujii, Koichi Yoshimura, Hiroki Aoki, Yuichi Orita, Takafumi Ishida, Megu Ohtaki, Masataka Nagao, Mari Ishida, Masao Yoshizumi

**Affiliations:** 1 Department of Cardiovascular Physiology and Medicine, Graduate School of Biomedical and Health Sciences, Hiroshima University, Hiroshima, Japan; 2 Department of Surgery and Clinical Science, Graduate School of Medicine, Yamaguchi University, Ube, Japan; 3 Cardiovascular Research Institute, Kurume University, Kurume, Japan; 4 Department of Cardiovascular Medicine, Graduate School of Biomedical and Health Sciences, Hiroshima University, Hiroshima, Japan; 5 Department of Environmetrics and Biometrics, Research Institute for Radiation Biology and Medicine, Hiroshima University, Hiroshima, Japan; 6 Department of Forensic Medicine, Graduate School of Biomedical and Health Sciences, Hiroshima University, Hiroshima, Japan; Yokohama City University Graduate School of Medicine, JAPAN

## Abstract

Abdominal aortic aneurysms (AAAs), which commonly occur among elderly individuals, are accompanied by a risk of rupture and subsequent high mortality. Establishment of medical therapies for the prevention of AAAs requires further understanding of the molecular pathogenesis of this condition. This report details the possible involvement of Osteoprotegerin (OPG) in the prevention of AAAs through inhibition of Tumor necrosis factor-related apoptosis-inducing ligand (TRAIL). In CaCl_2_-induced AAA models, both internal and external diameters were significantly increased with destruction of elastic fibers in the media in *Opg* knockout (KO) mice, as compared to wild-type mice. Moreover, up-regulation of TRAIL expression was observed in the media by immunohistochemical analyses. Using a culture system, both the TRAIL-induced expression of matrix metalloproteinase-9 in smooth muscle cells (SMCs) and the chemoattractive effect of TRAIL on SMCs were inhibited by OPG. These data suggest that *Opg* may play a preventive role in the development of AAA through its antagonistic effect on Trail.

## Introduction

An abdominal aortic aneurysm (AAA) is a progressive dilation of the aorta, which results in rupture with high mortality ratio. The infiltration of macrophages and disruption of medial elastic fibers that occur in this condition are believed to be associated with inflammation [1]. Since inflammation is a common underlying feature of both AAAs and atherosclerosis, these two conditions are considered to be closely related [2]. It has not yet been determined, however, whether the relationship between AAAs and atherosclerosis is causal or if these conditions merely share common risk factors, such as smoking, hypertension, and obesity [3]. Studies of human AAA tissue have shown extensive inflammatory infiltrates into both the tunica media and tunica adventitia, while atherosclerosis is primarily found within the tunica intima and tunica media. It has been suggested that AAA vascular tissue has a higher level of proteolytic activity, and a greater rate of inflammatory cell infiltration, as compared to atherosclerosis [4,5]. Proteolytic activities involved in the degradation of elastin in the supporting lamina of the aortic media depend on matrix metalloproteinase (MMP)-2 and MMP-9, as well as tissue inhibitor of metalloproteinase (TIMP)-1, the expression of which is elevated in the aneurysm tissues of both human and animal models [6–12]. MMPs are reportedly produced from macrophages and smooth muscle cells through the c-Jun NH2-terminal kinase (JNK) pathway [11,13]. These recent studies collectively suggest the involvement of several pathways for development of AAAs, although the pathogenesis of this condition has yet to be fully understood.

Osteoprotegerin (OPG, also referred to as TNFSF11B), a member of tumor necrosis factor (TNF) superfamily, has been shown to regulate the functions of many key genes for biological processes [14]. OPG was first identified as a main regulator of bone metabolism [15]. This soluble decoy receptor for the receptor activator of nuclear factor-kappaB ligand (RANKL, or TNFSF11) blocks the promotion of osteoclast differentiation and activation, which leads to bone resorption, by inhibiting RANKL binding to the activator of nuclear factor-kappaB (RANK, or TNFSF11A) [16]. OPG also works as a decoy receptor for the TNF-related apoptosis-inducing ligand (TRAIL, or TNFSF10) [14]. By signaling through death receptor-4 (DR4, or TNFRSF11A) and death receptor 5 (DR5, or TNFRSF11B), TRAIL is involved in two overlapping modes of signaling for cell death and cell survival, including transcriptional activation in normal tissues [17]. The function of this ligand in immune surveillance for tumors through the control of cell death signaling is well characterized. In the vascular system, studies using animal models have suggested that OPG plays a role in preventing arterial calcification and stabilizing plaque formation [18–23]. However, loss of the *Opg* gene has been reported to reduce Angiotensin II (AngII)-induced aneurysm formation in *ApolipoproteinE (ApoE)*-KO mice [24]. Clinical studies have shown positive correlations between serum OPG and the presence and progression of cardiovascular disease, including AAA [25,26]. However, whether the concentration of OPG in AAA tissue is up-regulated or down-regulated remains controversial [27,28]. Thus, while OPG may play an important biological role in the vascular system, its function remains ambiguous.

This study reports that loss of the *Opg* gene results in deterioration of AAAs, possibly through involvement of TRAIL in smooth muscle actin (SMA)-positive cells, including smooth muscle cells (SMCs) and myofibroblasts, in a CaCl_2_-induced AAA experimental model [13]. TRAIL has been shown to serve as a chemoattractant for SMCs and macrophages, as well as an inducer of *Mmp-9* and *Timp-1* transcription via the JNK and/or Nuclear factor-kappaB (NF-kB) pathways, leading to thickening of the medial layer and denaturation of the extracellular matrix (ECM) in the aortic wall. Thus, OPG may play an important role in the prevention of AAA formation via suppression of TRAIL signaling.

## Materials and Methods

### Generation of a Mouse Model for AAAs

Seven-week-old male wild-type and *Opg*-KO mice of the C57BL/6J strain (CLEA Japan, Inc.) underwent AAA induction via peri-aortic application of 0.5M CaCl_2_. For the control (sham) group, saline was used instead of CaCl_2_ (S1 Fig). After AAA induction, a time-course aortic analysis (from day one to week six) was performed. Under anesthesia, mice were perfusion-fixed with 4% paraformaldehyde in phosphate buffered saline (PBS) at physiological perfusion pressure. Abdominal aortas were then excised from the mice for histological examinations after images were taken. For RNA isolation, aortas were harvested after perfusion with PBS and stored in RNAlater solution (Ambion). This experimental protocol was approved by the Committee of Animal Experimentation at Hiroshima University (A08-32) and carried out in accordance with this protocol. All surgery was performed under sodium pentobarbital anesthesia, and all efforts were made to minimize suffering during and after surgery. At the end of each experiment, animals were sacrificed either by anesthetic overdose or intracardiac perfusion under anesthesia.

### Serum *Opg* Analysis

Serum Opg and Trail concentrations in wild-type mice at six weeks after AAA induction were measured using an ELISA kits (R&D Systems and RayBiotech).

### Morphological Examination

Paraffin-embedded aortic tissues were used to create 6-μm thick sections, which were subsequently stained with hematoxylin & eosin (HE), Elastica van Gieson (EVG), Aniline Blue-Azan, and von Kossa, using standard protocols. The maximum aortic diameter, which included thickened adventitia with inflammation, was measured as the external aortic diameter. For widths of the medial layers of the aorta, the maximum values on the sides to which CaCl_2_ was applied were measured. The averages of the maximum and minimum measurements of the internal cross-section of the aorta were calculated for the internal diameters of the aorta. All measurements were performed using Photoshop (Adobe Systems) and Image-J (NIH).

### Immunohistochemistry

Prior to staining, sections were preincubated in antigen-retrieval solution (pH 5.2) at 90°C for 45 minutes, based on the manufacturer’s instructions (Dako). For double-immunohistochemistry, antigen-retrieved sections were blocked with 1% bovine serum albumin in 0.1%Tween-PBS, incubated with a combination of primary antibodies, and subsequently incubated with appropriate secondary antibodies, including Alexa Fluor 488 or 555 dyes conjugated anti-mouse, -rat, -rabbit, or -goat antibodies (donkey, 1:500; Life Technologies). Sections were then counterstained with 4'-6-diamidino-2-phenylindole (DAPI). Antibodies for Alpha-SMA (mouse, monoclonal, 1:100; Sigma Aldrich), F4/80 (rat polyclonal, 1:100; Santa Cruz), Mmp-9 (goat polyclonal, 1:40; R&D Systems), Timp-1 (goat polyclonal, 1:50; LSBio), Trail (rabbit polyclonal, 1:100; Abcam), Vimentin (rabbit polyclonal, 1:50; BioVison) phosphorylated-SAPK/JNK (rabbit, 1:100; Cell Signaling), pNF-kB p65 (rabbit, 1:100; Cell Signaling), and Caspase-9 (rabbit, 1:50; Cell Signaling) were used as primary antibodies. Signals were detected by DMI4000 fluorescence microscope (Leica Microsystems). Photoshop (Adobe Systems) or Image-J (NIH) software was used to calculate the signal area, which was automatically detected based on color, and the entire intima-medial area, selected based on its morphology. The % of expression area of Trail, Mmp9, and Timp1 was then calculated by dividing the signal area by the entire intima-medial area.

### Real-Time PCR Detection

Total RNA was isolated using TRIzol reagent (Invitrogen). Reverse transcription was performed using ReverTra Ace qPCR RT Kits (TOYOBO). Real-time PCR was conducted using SYBR Premix Ex Taq II (Takara Bio Inc. and Kapa Biosystems Inc.). Intensities of PCR products were measured and analyzed using Opticon (MJ Research). The following primers were used for *Opg*: forward 5’-CCTGGAGATCGAATTCTGCTTGA-3’ and reverse 5’-TTTGCAAACTGTGTTTCGCTCTG-3’, *Trail*: forward 5’-ACGTTTAGACCATAGGCAACTGGA-3’ and reverse 5’-AGATTCAGTTAAAGCCTGCTGCTC-3’, *RANKL*: forward 5’-CATGTGCCACTGAGAACCTTGAA-3’ and reverse 5’-CAGGTCCCAGCGCAATGTAAC-3’, *Mmp-9*: 5’-GCCCTGGAACTCACACGACA-3’ and reverse 5’-TTGGAAACTCACACGCCAGAAG-3’. *Timp-1*: forward 5’-TGAGCCCTGCTCAGCAAAGA-3’ and reverse 5’-GAGGACCTGATCCGTCCACAA-3’, and *Mmp-2*: forward 5’-CTCCTACAACAGCTGTACCAC-3’ and reverse 5’-CATACTTGTTGCCCAGGAAAG-3’. Amplification conditions were 5 s at 95°C, 20 s at 60°C, and 15 s at 72°C for 49 cycles. Internal controls included *G3pdh* or *β-actin*.

### Cell Culture

Mouse aortic SMCs were isolated from the abdominal aorta (from the diaphragm to the bifurcation) in 5-week-old wild-type male mice as previously described [29], and maintained in Dulbecco's Modified Eagle Medium (DMEM) with 10–20% fetal bovine serum (FBS). Cells were growth arrested at 70–80% confluence via incubation in serum-free medium for 48 hours before stimulating cells with rh-TRAIL (CD253; AbD Serotec). Different concentrations of rh-OPG (Peprotech) were used to block TRAIL; MG-132 (Calbiochem) or BAY11-7082 (Calbiochem) was used for inhibition of NF-kB activity, and AS-601245 (Enzo Life Sciences) or SP600125 (Calbiochem) for inhibition of JNK activity two hours before TRAIL induction.

### Migration Assay

Migration assays were performed using the ThinCert cell culture insert (Greiner Bio One), a modified Boyden chamber equipped with a polycarbonate membrane filter with 8 μm pores. Mouse peritoneal macrophages were isolated from five-week-old male wild-type mice via intra-peritoneal injection of 3% Brewer thioglycollate medium (Sigma Aldrich) for four days prior to isolation. Samples were maintained and frozen in Roswell Park Memorial Institute (RPMI) medium, as previously described [30]. Mouse aortic SMCs, isolated from the abdominal aorta, or macrophage cells from intraperitoneal fluid were seeded on top of the membranes at a density of 1x10^5^ cells, with the bottom of the membrane immersed in 1 μg/ml of rh-TRAIL, 100 ng/ml of rh-MCP-1 (R&D Systems), or 0.5 μg/ml of PDGF (Sigma-Aldrich) in DMEM. Different concentrations of rh-OPG (Peprotech) were also added for inhibition of TRAIL-induced migration. After incubation of macrophages for 90 minutes or SMCs for six hours at 37°C in 5% CO_2_, tops of the membranes were wiped with an absorbent cotton to remove non-migratory cells and then stained with Diff-Quick staining solution (Sysmex). Migrated cells on the bottom surface of the membranes were counted in 10 microscopic fields as previously described [31,32].

### Western Blotting

Cell lysate was prepared from growth-arrested VSMCs, which were stimulated with rh-TRAIL and lysed in radio-immunoprecipitation assay (RIPA) lysis buffer [31,32]. Samples containing equal amounts of protein were separated by SDS-PAGE and transferred onto a nitrocellulose membrane. The membrane was incubated with a primary antibody (Mmp-9, goat polyclonal, 1:1000, R&D Systems; Timp-1, goat polyclonal, 1:1000, LSBio; OPG, rabbit polyclonal, 1:1000, Abcam) and subsequently with an appropriate secondary antibody. Signals were visualized using the ECL system (Amersham-Pharmacia Co, UK). Images were captured using Photoshop (Adobe Systems) and densitometry was performed with Image J software (NIH).

### Statistical analysis

One-way analysis of variance (ANOVA) with Fisher’s post-hoc analysis, was used (if not indicated otherwise), provided that Bartlett’s test (p<0.05) showed homogeneity of variances. A non-parametric analysis, the Mann-Whitney’s U-test or Kruskal-Wallis test with Scheffe’s and Steel-Dwass post-hoc analyses was used. These analyses were performed with Ekuseru-Toukei 2012 software (Social Survey Research Information Co., Ltd.). Multiple regression analyses with spline functions were also performed for external diameter, internal diameter, and medial layer. All data are expressed as mean ± standard deviation (SD). P<0.05 was considered statistically significant.

## Results

### Absence of *Opg* Gene-Enhanced AAA Formation

**Fig 1:**
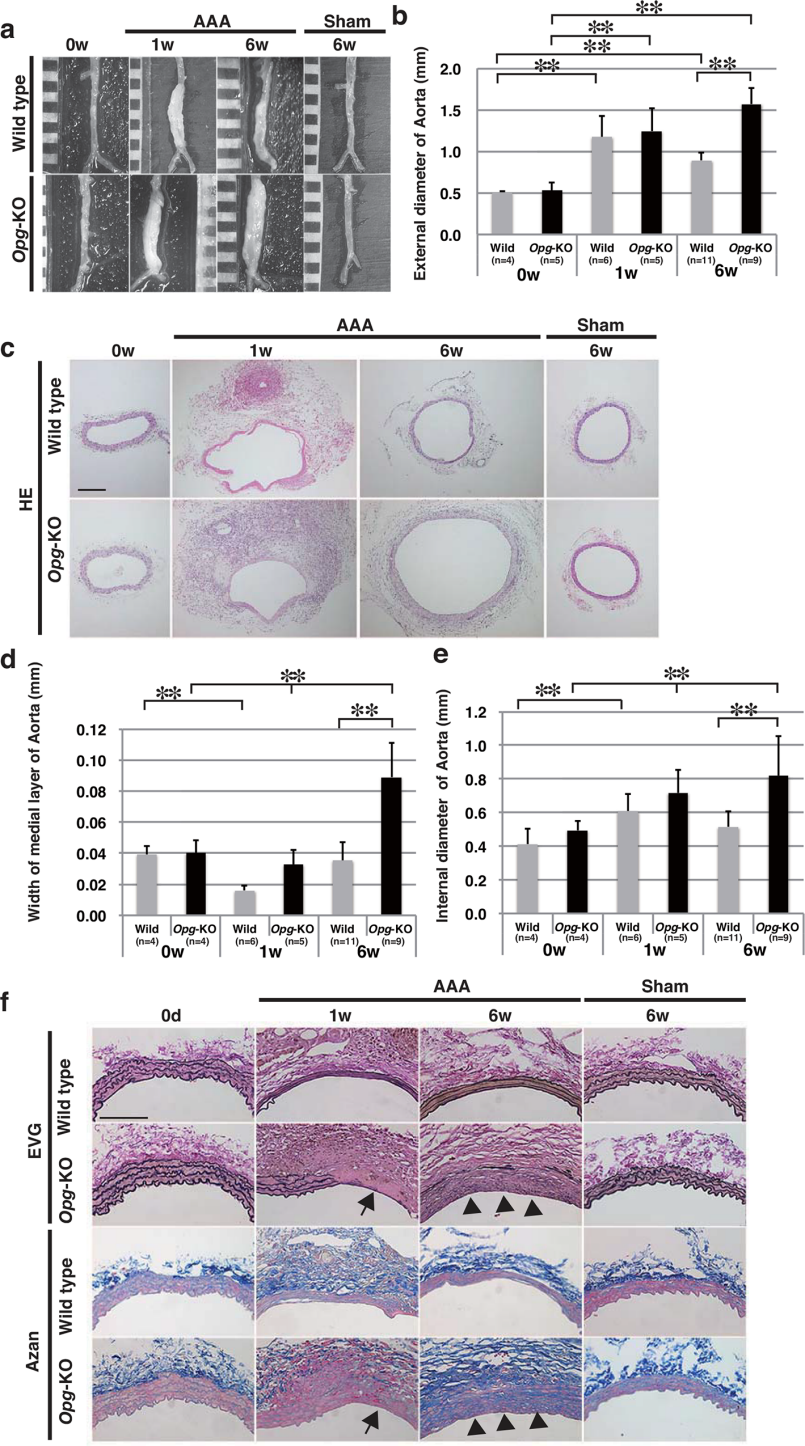
Prolonged Dilatation of the Abdominal Aorta in *Opg*-KO Mice. (a) Representative aortic images are shown at zero (0 w), one (1 w), and six weeks (6 w) after the application of CaCl_2_ (AAA) or saline (sham) in wild-type and *Opg*-KO mice. Scale bars indicate 1 mm. (b) Measurements of the external aortic diameter by weeks (w) after AAA induction. (c) Representative hematoxylin & eosin (HE) staining images are shown. Scale bars indicate 200 μm. (d, e) Measurements of the widths of medial layers (d) and the internal aortic diameter (e) are presented. Data are presented as mean ± SD. The number of samples for analysis is shown in parentheses; *p<0.05 or **p<0.01, as compared with the parametric multiple comparison procedure. (f) Representative aortic Elastica van Gieson (EVG) and Azan staining images are presented. Arrows show partial disruption at one week. Arrowheads show total disruption of elastic lamella (EVG) and irregular accumulation of collagen fibers (Azan) at six weeks after AAA induction in the aortic walls of *Opg-*KO mice. Scale bars indicate 100 μm.

We first tested whether the loss of *Opg* genes would affect AAA development, which was induced by application of CaCl_2_ to the abdominal aorta [13]. In wild-type mice, significant enlargement of the external aortic diameters was found at one week after AAA induction, and was retained at six weeks after induction (Fig 1A and 1B). In *Opg*-KO mice, the external aortic diameter was also enlarged at one week, and showed gradual dilation afterward. At six weeks after AAA induction, the external aortic diameters of *Opg*-KO mice were larger than those of wild-type mice. Sham operations did not result in significant changes at any point (S1 Fig). Hematoxylin and eosin (HE) staining of sections showed thinning of the medial layer and enlargement of the internal diameter at one week in both wild-type and *Opg*-KO mice (Fig 1C, 1D and 1E). However, at six weeks, thickening of the medial layer and dilatation of the internal diameter were evident in *Opg*-KO mice (Fig 1C, 1D and 1E). Through the application of multiple regression analyses with spline functions, statistically significant increases in the external diameter, medial layer, and internal diameter were also detected in *Opg*-KO mice at six weeks (S2 Fig).

Elastica van Gieson (EVG) and Azan staining showed typical wavy lamellae of elastic and collagen fibers at regular intervals in the medial layer of wild-type and *Opg-*KO mice at day zero and at six weeks after the sham operation (Fig 1F). However, after AAA induction, elastic fibers appeared to have flattened lamellae at thinner intervals, but never disappeared in wild-type mice. In contrast, partial or entire disappearance of the medial elastic lamellae and accumulation of collagen fibers were observed in *Opg-*KO mice (Fig 1F). These observations suggest that aortic properties of the extracellular matrix, especially in the media, were severely altered in *Opg-*KO mice.

### Up-Regulation of *Opg* Expression in Response to AAA Formation

**Fig 2:**
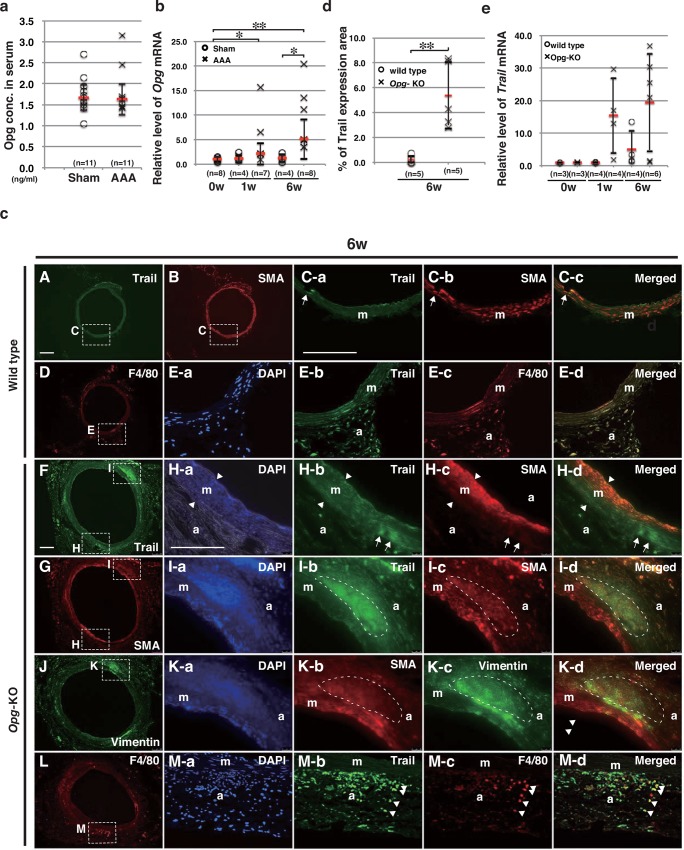
Enhanced Up-Regulation of Trail in the Media of *Opg-*KO Mice. (a) Serum *Opg* concentration was measured by enzyme-linked immunosorbent assay (ELISA) at six weeks after either sham or AAA operations in wild-type mice. No significant difference between sham and AAA was found by the Mann-Whitney’s U-test. (b) *Opg* mRNA expression in the abdominal aorta at one or six weeks after sham or AAA operations in wild-type mice. (c) Representative double immunofluorescent staining shows Trail (A, F) in green, SMA (B, G) in red, F4/80 (D, L) in red, and Vimentin (J) in green, in the aortas of wild-type (A, B, D) and *Opg*-KO (F, G, J, and L) mice. SMA and F4/80 signals, indicated by rectangles in panels A, B, D, F, G, J, and L, are magnified and merged with the expression of Trail in green (H-b to H-d, I-b to I-d, and M-b to M-d). Vimentin signal, indicated by rectangles in panel J, is magnified and merged with SMA in red (K-b to K-d). Nuclei are stained with DAPI in blue (E-a, H-a, I-a, K-a and M-a). Arrowheads in panels H-a to H-d indicate the width of the medial layer. Arrows in panels H-b to H-d show strong Trail expression in weakly SMA-positive cells in the media. The region surrounded by the white dotted line in panels I-b to I-d indicate strong Trail expression in weakly SMA-positive cells in the adventitia. These weakly SMA-positive cells in the adventitia also express vimentin (K-c). Arrowheads in panels M-b to M-d indicate F4/80 and Trail double positive cells. m: medial layer; a: adventitia. Scale bars represent 100 μm. (d) Percentage of the Trail-expressing area in the medial layer of the aorta at six weeks after AAA induction in wild-type or *Opg*-KO mice. Representative photographs are shown in S4 Fig. (e) *Trail* mRNA levels in the aorta at one or six weeks after AAA induction in wild-type or *Opg*-KO mice. Data are presented as mean ± SD. The number of samples for analysis is shown in parentheses; *p<0.05 or **p<0.01, as compared to 0w or wild-type using the Kruskal-Wallis test.

To investigate the role of Opg in AAA formation, its expression was measured in the serum and aortic tissue of wild-type mice. There was no significant difference in serum Opg concentration between sham operation and AAA operation groups (Fig 2A). Instead, the expression level of *Opg* mRNA in the aorta was up-regulated in response to AAA induction in wild-type mice (Fig 2B). In order to determine whether Opg is produced by aortic SMCs as previously reported [33], we performed western blot analysis and found that Opg protein was only detected in lysates of cultured SMCs from wild-type mice, but not from the *Opg-*KO mice, with or without rh-TRAIL (S3A Fig). These results suggest that aortic SMCs are the primary source of Opg and its expression increases during development and progression of AAAs.

### Up-Regulation of Trail in the media of AAAs in *Opg*-KO Mice

Given the role of OPG as a decoy receptor for TRAIL, we first tested whether its expression is up-regulated in aortic tissue. Although the aortic expression of *Trail* mRNA at six weeks after AAA induction tended to be higher in *Opg-*KO mice than in wild-type mice, the difference was not significant (Fig 2E). Moreover, no difference was found in the serum concentration of Trail between wild-type and *Opg-*KO mice at six weeks after AAA induction (data not shown).

We next determined whether Trail is up-regulated in SMCs, or macrophages, by double-immunofluorescent staining using antibodies that recognize SMA and macrophages (F4/80). At six weeks after AAA induction, Trail was detected weakly in most SMCs of the media of *Opg-*KO mice, but strongly in some SMCs, particularly those located at the peripheral edge of the medial layer and elastic fibers, in both wild-type (Fig 2C-A, 2C-B and 2C-C and S4A–S4E Fig) and *Opg-*KO mice (Fig 2C-F, 2C-G and 2C-H and S4F–S4J Fig). This suggests that Trail is expressed in SMCs. Trail was also expressed in some SMA-positive cells in the adventitia (Fig 2C-F, 2C-G and 2C-I). However, vimentin was also present in almost all SMA-positive cells in the adventitia, suggesting that these cells might be myofibroblasts (Fig 2C-J and 2C-K). Trail expression was also observed in large, F4/80-positive round-shaped macrophages in the adventitia in both wild-type (Fig 2C-D and 2C-E) and *Opg*-KO mice after AAA induction (Fig 2C-L and 2C-M). This suggests that Trail is expressed in SMCs, macrophages, and myofibroblasts in the aorta. In order to determine whether Trail would be up-regulated in the absence of OPG, we measured the Trail-positive area in the media (S4 and S6 Figs) and found that this area in *Opg*-KO mice was significantly larger than that in wild-type mice (Fig 2D).

Since TRAIL enhances apoptosis in cancer [17], we determined whether apoptosis would be detected after AAA induction by immunohistochemical detection of Caspase-9, a marker of apoptosis. Caspase-9 was not detected in the aortic wall six weeks after AAA induction in either wild-type or *Opg-*KO mice, suggesting that Trail may not be involved in apoptosis in this context (S5 Fig). We also tested whether TRAIL could induce SMC proliferation, given previous reports that it enhances SMC proliferation [34]. An increase in the BrdU-positive SMCs was observed after AAA induction in the medial layer and adventitia of *Opg-*KO mice compared to wild-type mice (data not shown). However, rh-TRAIL did not induce significant growth, despite a trend for an increased number of cells in SMCs culture system. Thus, whether Trail enhances SMC proliferation during AAA formation remains inconclusive.

### Trail is a Chemoattractant for SMCs and Macrophages

**Fig 3:**
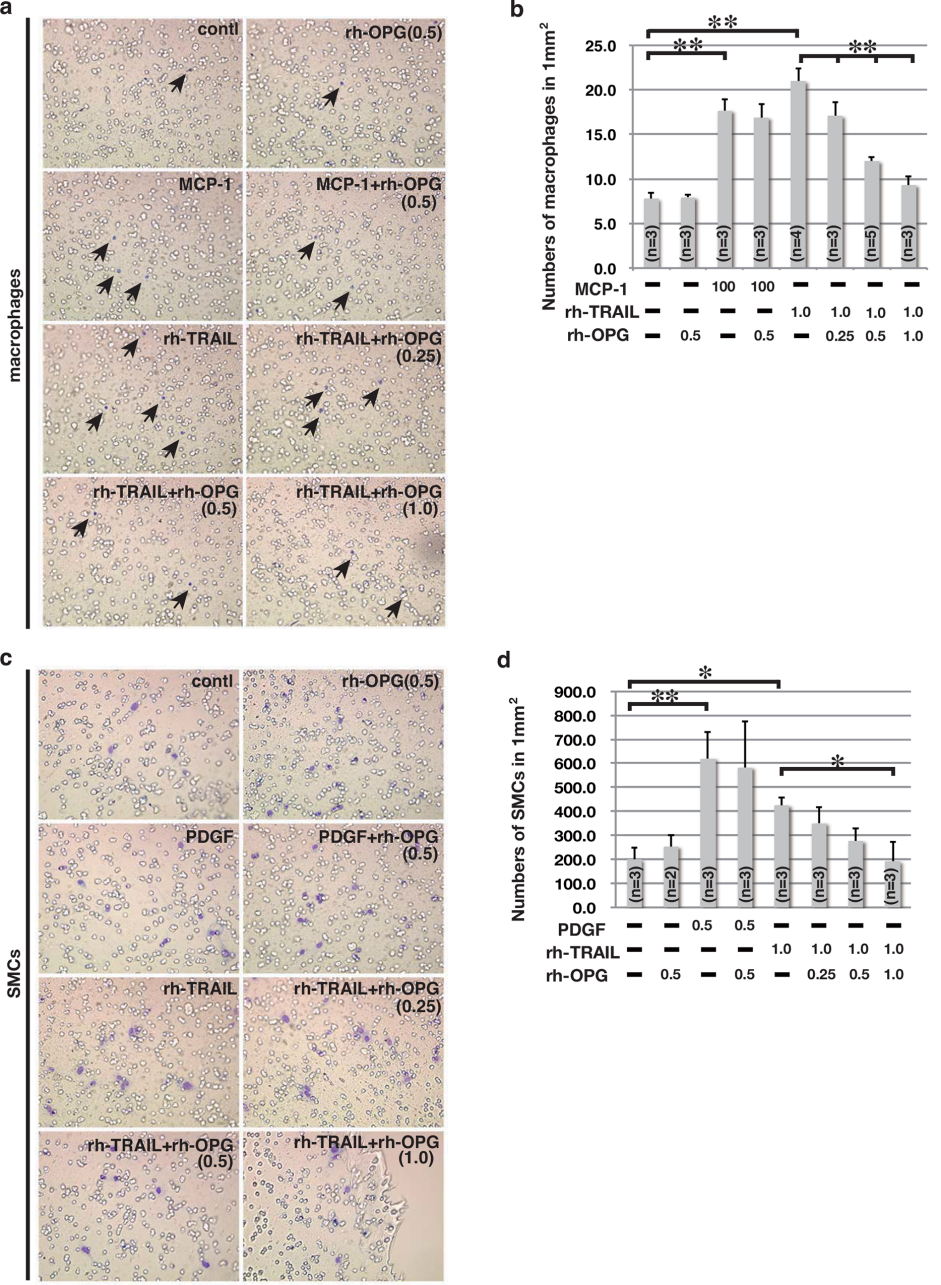
Chemoattractive Effects of TRAIL on Peritoneal Macrophages and Aortic SMCs. (a, c) Representative images of membranes stained with Diff-Quick solution. Peritoneal macrophages (arrows in panel a) and aortic SMCs (c) that have migrated to the membrane surface are seen in violet. Chambers were immersed in monocyte chemotactic protein-1 (MCP-1) (100 ng/ml), platelet-derived growth factor (PDGF) (0.5 μg/ml), or rh-TRAIL (1.0 μg/ml). The concentration of rh-OPG was 0.25–1.0 μg/ml. (b, d) Measurements of macrophages (b) or aortic SMCs (d) that have migrated per one mm^2^ of membrane area. Data are presented as mean ± SD (at least 3 independent experiments were performed); *p<0.05 or **p<0.01, as compared to controls or TRAIL alone.

As TRAIL has been reported to induce chemotactic migration of monocytes toward the site of inflammation [31], it was hypothesized that TRAIL also functions as a chemoattractant for SMCs. Since Trail was detected in the elastic fibers and the peripheral edge of the medial layer (Fig 2C), this property could be responsible for the accumulation of SMCs in the media. A cell culture-based migration assay confirmed that rh-TRAIL could induce migration of mouse abdominal macrophages, with dose-dependent repression by rh-OPG application (Fig 3A and 3B). Similarly, rh-TRAIL induced the migration of mouse aortic SMCs, with dose-dependent inhibition by rh-OPG (Fig 3C and 3D). These results suggest that the release of a larger quantity of TRAIL induced a greater amount of SMC migration in *Opg-*KO mice.

### Trail Can Induce Its Own Expression

Due to the continuous up-regulation of Trail expression in the absence of *Opg*, it was hypothesized that *Trail* could induce its own expression. Within a SMC culture system, incubation with rh-TRAIL induced up-regulation of the expression of *Trail* mRNA, but not *Opg* mRNA (S3B and S3C Fig). *Trail* up-regulation was inhibited by pre-incubation with rh-OPG, whereas rh-OPG had no effect on *Trail* expression (S3D and S3E Fig). These results suggest that TRAIL-induced *Trail* mRNA production may be a cause of continuous *Trail* up-regulation in the development of AAAs in *Opg*-KO mice.

### *Mmp-9* and *Timp-1* Co-localize with Trail in the Media of AAA

**Fig 4:**
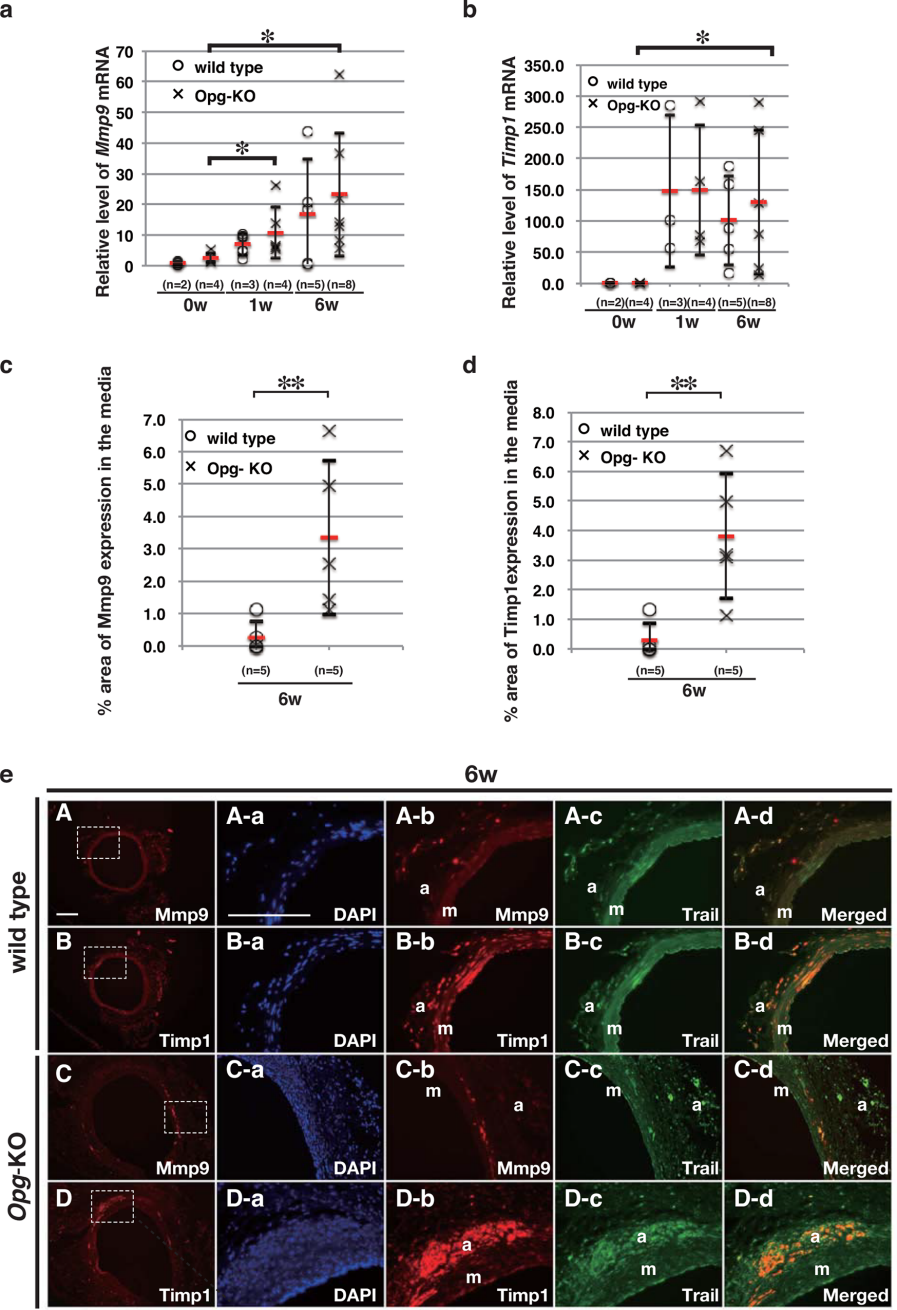
Imbalanced Accumulation of *Mmp-9* and *Timp-1* in the Media of *Opg*-KO Mice. (a-b) Relative expression levels of *Mmp-9* (a) and *Timp-1* (b) mRNA in the abdominal aorta at one or six weeks after AAA induction in wild-type or *Opg*-KO mice. (c, d) Percentage of Mmp-9 (c) and Timp-1 (d) expressing areas in the medial layer of the aorta was measured in three slides from each sample at six weeks after AAA induction in wild-type or *Opg*-KO mice. Data are presented as mean ± SD (at least 3 independent experiments were performed). The number of samples for analysis is shown in parentheses; *p<0.05 or **p<0.01, as compared to expression before the operation or wild-type using the Kruskal-Wallis test. (e) Representative double immunofluorescent staining images, with Trail in green, Mmp-9 in red, and Timp-1 in red. Images were taken in the abdominal aorta at six weeks (A-D) after AAA induction in wild-type (A, B) or *Opg*-KO (C, D) mice. Mmp-9 and Timp-1 signals, indicated by rectangles in panels A through D, are magnified and merged with Trail expression in green (Aa-d, Ba-d, Ca-d, Da-d). Nuclei are stained with DAPI in blue (m: medial layer; a: adventitia). Scale bars represent 100 μm.

Since imbalanced production of MMPs and TIMPs has been suggested to cause changes in medial elastic fibers, we assessed whether *Mmp-9* and *Timp-1* mRNA levels were altered in the AAA model. *Mmp-9* mRNA expression was up-regulated in the aorta of wild-type and *Opg*-KO mice, as assessed by qRT-PCR (Fig 4A). *Timp-1* mRNA expression was continuously up-regulated in both wild-type and *Opg*-KO mice after AAA induction (Fig 4B). In order to determine whether Mmp-9 and Timp-1 are up-regulated in the media, we measured the Mmp-9- and Timp-1-positive areas in the media by immunofluorescent staining (S6A and S6B Fig). The Mmp-9- and Timp-1-expressing areas in *Opg*-KO mice were significantly larger than those in wild-type mice (Fig 4C and 4D).

Next, to determine whether Trail regulates Mmp-9 and Timp-1 expression, we performed double immunohistochemical analysis. At six weeks, the expression of Mmp-9 was reduced in wild-type mice (Fig 4EA), while Timp-1 was still expressed with Trail in the medial layer as well as the adventitia (Fig 4EB). In contrast, in *Opg*-KO mice, focal expression of Mmp-9 was still observed in the medial layer (Fig 4EC), and Timp-1 was expressed mainly in the adventitia (Fig 4ED). These results indicate that focal expression of Mmp-9 in the medial layer of *Opg*-KO mice, regardless of the continuous expression of Timp-1, could be the main cause of elastic fiber disappearance and might be regulated by Trail.

### *Mmp-9* and *Timp-1* Are Up-Regulated via the JNK and NF-kB Signaling Pathways

**Fig 5:**
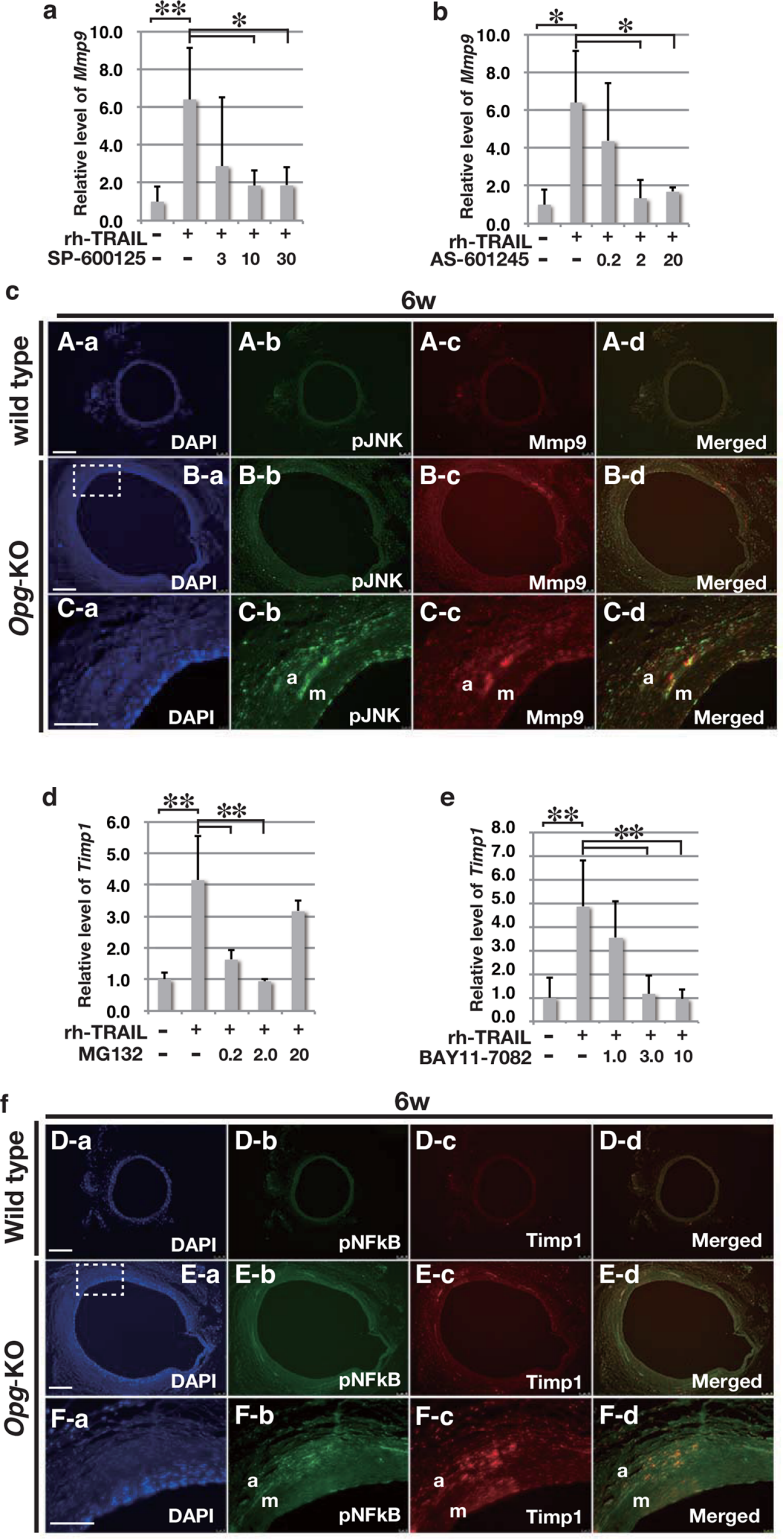
Induction of *Mmp-9* and *Timp-1* mRNA by Trail via the JNK or NF-kB Signaling Pathway. (a, b, d, e) Relative expression levels of *Mmp-9* (a, b) and *Timp-1* (d, e) mRNA in mouse aortic SMCs within a culture system induced by rh-TRAIL (10 ng/ml). SP-600125 (3–30 μM) (a) and AS-601245 (0.2–20 μM) (b) were used to inhibit the JNK signaling pathway. MG-132 (0.2–20 μM) (d) and BAY11-7082 (1–10 μM) (e) were used to inhibit the NF-kB pathway. Data are presented as mean ± SD; *p<0.05 or **p<0.01, as compared to controls or TRAIL alone. Samples were in triplicate for each group. (c, f) Representative double immunofluorescent staining images of abdominal aortas in wild-type and *Opg-*KO mice at six weeks, with pJNK (panels A-b, B-b, and C-b in c) or pNF-kB (panels D-b, E-b, and F-b in f) in green, and Mmp-9 (panels A-c, B-c, and C-c in c) or Timp-1 (panels D-c, E-c, and F-c in f) in red. pJNK signal merged with Mmp-9 (panels A-d, B-d, and C-d in c). pNF-kB signal merged with Timp-1 (panels A-d, B-d, and C-d in f). Rectangles in panels B and E show magnified areas in C and F, respectively. Nuclei are stained with DAPI in blue (m: medial layer; a: adventitia). Scale bars in panels A, B, D, and E represent 100 μm. Scale bars in panels C and F represent 50 μm.

TNF-α has been reported to up-regulate *Mmp-9* expression via the JNK signaling pathway [35]. Given that TRAIL is a member of the TNF superfamily, we tested whether Trail induces *Mmp-9* expression. Within a SMC culture system, Mmp-9 was up-regulated by rh-TRAIL (S7A and S7C Fig). This up-regulation of *Mmp-9* was inhibited by rh-OPG (**S7B Fig**), as well as JNK inhibitors SP-600125 and AS-601245 (Fig 5A and 5B). The distribution of Mmp-9 overlapped with that of phosphorylated JNK in the adventitia (Fig 5C). *Mmp-2* expression was also up-regulated by rh-TRAIL via the JNK signaling pathway (S7D and S7E Fig).

TRAIL also induced *Timp-1* expression, which was inhibited by rh-OPG (S7C, S7F and S7G Fig). TRAIL-induced *Timp-1* mRNA expression was blocked by inhibitors of the NF-kB signaling pathway, MG-132 and BAY11-7082 (Fig 5D and 5E). Timp-1 distribution overlapped with phosphorylated NF-kB (Fig 5F), rather than with phosphorylated JNK (data not shown), suggesting that Trail may induce the expression of *Timp-1* via the NF-kB signaling pathway *in vivo*.

## Discussion

In this study, we found that dilation of the abdominal aorta was enhanced with complete destruction/disappearance of elastic fibers in the medial layer of *Opg-*KO mice by using a CaCl_2_-induced AAA model. Continuous Trail expression was observed in the medial layer of *Opg-*KO mice and overlapped with the expression of Mmp-9 and Timp-1. In the SMC culture system, TRAIL induced the expression of *Mmp-9* and *Timp-1* mRNA. Induction of Mmp-9 by TRAIL occurred via the JNK pathway, and Timp-1 was induced via NF-kB and the JNK pathway. Given that Mmp-9 is known to be the primary cause of elastic fiber disruption [9] [36] [37] [38], extracellular matrix remodeling in the medial layer could be associated with dilation of the abdominal aorta. Previous studies have also shown that MMP9 is induced via activation of the JNK pathway in AAA models [13] and that TRAIL has non-apoptotic functions, such as activation of JNK and NF-kB signaling in HeLa and human embryonic kidney 293 cell lines [39,40], although no downstream target genes related to these non-apoptotic aspects have been identified. In the present study, we identified downstream targets of Trail, which is under the control of Opg, suggesting that the Opg-Trail cytokine system could be implicated in the maintenance of medial layer integrity.

Accumulation of myofibroblasts was apparent in the adventitia of *Opg*-KO mice at six weeks after CaCl_2_ treatment. Trail distribution was widely observed in myofibroblasts and co-localized with Mmp9 and Timp1 at one week after treatment (data not shown). However, Trail expression later shifted to a region where cells exist at high density with up-regulated expression of Timp1 in the adventitia. We speculate that Timp1 expression causes ECM accumulation, depriving myofibroblasts of their mobility. In wild-type mice, the accumulation of myofibroblasts in the adventitia was also seen after one week of CaCl_2_ treatment, but was attenuated afterwards. On the other hand, in *Opg*-KO mice, proliferation and accumulation of myofibroblasts lasted up to six weeks after CaCl_2_ treatment (data not shown). Myofibroblasts are mainly involved in ECM production and modification, secretion of angiogenic and pro-inflammatory factors, and generation of tensile force [41]. Thus, Opg may also play an important role in maintaining vascular integrity in the adventitia.

In the present study, *Opg* mRNA levels were up-regulated in the aortic tissue of wild-type mouse AAAs. Our results are consistent with human studies showing an increased tissue concentration of OPG in AAA [27,28]. Recently, Moran et al. reported that aortic dilation and rupture was limited in Angiotensin (Ang) II-treated *ApoE/Opg* double KO (dKO) mice, compared to those of similarly treated *ApoE* single KO mice, suggesting that Opg could attenuate AAA formation in their model system [24]. One possible explanation for the discrepancy between their results and ours is the different amount of circulating AngII. With continuous infusion of AngII, Opg may accelerate the progression of AAA. However, without AngII addition, Opg may prevent AAA formation through our proposed mechanism. This may suggest that OPG functions as a double-edged sword depending on surrounding conditions. The important role of AngII in the formation of AAA provides us with insight regarding the use of AngII blockers in humans.

In animal studies, the function of Opg in the formation of arteriosclerosis has been a source of controversy. A few studies have demonstrated the promotion of arteriosclerosis by Opg [42,43], yet most studies have reported a preventive role of Opg in cardiovascular disease models [18–21,42]. Clinical studies have shown elevated serum OPG levels in cardiovascular diseases[25–28,44], and it is suggested that increased serum OPG levels in humans may be caused by a self-defensive compensatory response to inflammation. In the present study, while *Opg* mRNA levels were up-regulated in the aortic tissue of wild-type mouse AAAs, serum levels remained the same. In humans, the entire vasculature is involved in inflammation, whereas in a mouse model, lesion areas induced by CaCl_2_ application are relatively small. This may be a reason why serum Opg levels remained unchanged in the present wild-type mouse AAA model. Our results support the preventive role of Opg in AAA pathogenesis.

Based on this study, a model for AAA induction is proposed (**S8 Fig**). After focal application of CaCl_2_, some SMCs are released from the medial layer. In wild-type mice, residual SMCs expressed Trail, which then induced *Mmp-9* and *Timp-1* expression. The balanced enhancement of these factors may result in the flattening of wavy lamella structure within the media, which resembles vascular remodeling [45]. Until six weeks, SMCs express Opg, attenuate the function of Trail, and continue to proliferate in the medial layer (data not shown), resulting in recovery of the medial layer. In contrast, the loss of *Opg* may lead to continuous *Trail* mRNA expression in SMCs via an autocrine mechanism. Prolonged up-regulation of both *Mmp-9* and *Timp-1*, causing a local imbalance, could result in complete destruction of the elastic lamella. After this loss of elastic lamella, proliferation of SMCs was delayed but continued in the medial layer (data not shown), leading to thickening of the medial layer. Thus, in the aortas, OPG may function as an anti-inflammatory molecule.

In the adventitia, small populations of SMCs might migrate and attract macrophages by secreting Trail. The accumulation of SMA/Vimentin-positive cells might be attributed to continuous Trail secretion from SMCs and macrophages, or myofibroblasts derived from macrophages, as well as fibroblasts [46]. This process may be accompanied by a significant increase in adventitia volume. Taken together, Trail could be considered a cytokine that triggers inflammatory responses. Opg, as an antagonist of Trail, may prevent excessive inflammation. Thus, Opg and Trail together may contribute to the integrity of the vascular wall. In humans, OPG may also function as an inhibitor of TRAIL; if this inhibitory effect is insufficient, formation of AAAs may be accelerated. Further studies are necessary to investigate the function of OPG in the human vascular wall.

## Supporting Information

S1 Fig Aortic Diameter Was Unchanged by Sham Operations. **(a)** Representative images of the aortic samples with corresponding number of days (d) and weeks (w) since saline application (sham operation) in wild-type and *Opg*-KO mice. Scale bars indicate one mm. **(b-d)** Measurements of external diameter (**b**), width of medial layers (**c**) and internal diameter (**d**) with corresponding number of days (d) and weeks (w) since sham operations in wild-type and *Opg*-KO mice. The number of samples for analysis is shown in parentheses.
(EPS)

S2 FigAortic Diameter Was Significantly Increased in *Opg*-KO Mice AAA by Multiple Regression Analyses.**(a-c)** Measurements and fitted time-trends of external diameters (a), medial layers (b), and internal diameters (c) of abdominal aortas in wild-type and *Opg*-KO mice at six weeks after AAA induction. **(a)** Given the external diameter measurement data {(*y*_*i*_, *t*_*i*_, *s*_*i*_), *i* = 1,…, *n*}, where *s*_*i*_ = 1 if (OPG-KO), -1 if (wild-type), the following regression model was applied: log(*y*_*i*_) = *β*_0_ + *β*_1_ log(1 + *t*_*i*_) + *β*_2_{log(1 + *t*_*i*_) − *τ*}^2^ + *β*_3_*s*_*i*_ × [log(1 + *t*_*i*_) − *τ*]_+_ + *ε*_*i*_, i = 1,…, *n*. where [*z*]_+_ = (*z*+ | *z* |) / 2, *β*_0_, *β*_1_, *β*_2_, *β*_3_ and *τ* are unknown parameters to be estimated. The terms *ε*_*i*_, *i* = 1,…, *n*, represent errors, which are mutually independent normal distributions with means of zero and variances of *σ*^2^. These unknown parameters were estimated with the least square method. The resultant estimates are: ${\hat{\beta }}_{0}=0.2333,\;{\hat{\beta }}_{1}=-0.0040,\;{\hat{\beta }}_{2}=-0.0993,\;{\hat{\beta }}_{3}=0.3795,\;\text{and}\;\hat{\tau }=3.02.$ The difference in time-trends between the two groups, which is expressed by ${\hat{\beta }}_{3}=3.795,$ was highly statistically significant (*p* = 1.64×10^−11^). (**b**) Using a similar method to the analysis of external diameter data (a), the following regression model was applied to medial layer data: log(*y*_*i*_) = *β*_0_ + *β*_1_*t*_*i*_ + *β*_2_*s*_*i*_ + *β*_3_*s*_*i*_ × *t*_*i*_ + *ε*_*i*_, *i* = 1,…, *n*. The resulting unknown parameters were as follows: ${\hat{\beta }}_{0}=-3.698,$ ${\hat{\beta }}_{1}=0.0191,\;{\hat{\beta }}_{2}=0.1347,\;(p=0.0027),\;{\hat{\beta }}_{3}=0.0090\;(p=2.39\times 10^{-6}).$ (**c**) Using a similar method to that discussed above, the following regression model was applied to internal diameter measurement data: log(*y*_*i*_) = *β*_0_ + *β*_1_*t*_*i*_ + *β*_2_(*t*_*i*_ − *τ*)^2^ + *β*_3_*s*_*i*_×[*t*_*i*_ – *τ*]_+_ + *ε*_*i*_, *i* = 1,…, *n*. The resultant unknown parameters were as follows: ${\hat{\beta }}_{0}=-0.4613,$ ${\hat{\beta }}_{1}=0.00231,\;{\hat{\beta }}_{2}=-0.000379,\;{\hat{\beta }}_{3}=0.01296\;(p=5.34\times 10^{-5}).$(EPS)

S3 Fig Trail Induces Expression of *Trail* but Not *Opg* mRNA, Opg inhibits Trail-induced expression of *Trail* mRNA, and SMCs are a source of OPG. **A,** Western blotting analysis for Opg in cultured SMCs with or without rh-TRAIL (10 ng/ml). **(b-e)** Relative expression levels of *Trail* **(b, d, e)** and *Opg* mRNA **(c)** in a mouse aortic SMC culture system. Specific concentrations of rh-TRAIL (1–100 ng/ml) were used for induction of *Trail* **(b)** and *Opg* **(c)** expression. **(d)** *Trail* mRNA expression induced by rh-TRAIL (10 ng/ml) was inhibited by rh-OPG (5–50 ng/ml). **(e)** *Trail* mRNA expression was not affected by OPG alone (5–50 ng/ml). Samples were in triplicate for each group. Data are presented as mean ± SD, **p<0.01, *p<0.05 compared to control or rh-TRAIL alone.
(EPS)

S4 Fig Trail Expression Area in the Media is Larger in *Opg*-KO Mice. Representative images of double immunofluorescent staining of the aorta with Trail (**A-b to J-b**) in green and SMA (**A-c to J-c**) in red, at six week after AAA induction in wild-type (**A-E**) or *Opg*-KO (**F-J**) mice. Areas in red were measured as the entire intima-medial area. Areas of intensive green signal were measured as the Trail expression area. All samples used for analysis are listed with their identification numbers. Scale bars represent 100 μm.
(EPS)

S5 Fig Trail Does Not Induce Apoptosis in Aneuryzed Aorta. Representative images of double immunofluorescent staining of the aorta with Caspase-9 in green and SMA in red, at one or six weeks after AAA induction in wild-type or *Opg*-KO mice. Scale bars represent 50 μm.
(EPS)

S6 Fig *Trail* Expression Overlaps with Mmp-9 and *Timp-1* Expression. **(a, b)** Double immunofluorescent staining of the aorta with Trail in green (**A-E, K-O**), and Mmp-9 (**a**) or Timp-1 (**b**) in red (F-J and P-T), at six week after AAA induction in wild-type (**A-J**) or *Opg*-KO (**K-T**) mice. Selected areas, representatively shown in white in A-b, F-b, K-b, and P-b, were measured as the area of the intima-medial layer. In these selected areas, intensive signals in green or red were measured as expression areas for Trail and Mmp-9 or Timp-1, respectively. All samples used for analysis are listed with their identification numbers. Scale bars represent 100 μm.
(EPS)

S7 Fig Induction of *Mmp-9* and *Timp-1* by TRAIL is Inhibited by *Opg.* **(a, b, d-g)** Relative expression level of *Mmp-9* **(a, b)**, *Mmp-2* **(d, e)**, and *Timp-1* **(f, g)** mRNA in mouse aortic SMCs culture system. The indicated concentrations of rh-TRAIL (1–100 ng/ml) were used to induce *Mmp-9* **(a),** *Mmp-2* **(d)** and *Timp-1* **(f).** *Mmp-9* **(b)** as well as *Timp-1* **(g),** induced by rh-TRAIL (10 ng/ml), are inhibited by rh-OPG (5-50ng/ml). *Mmp-2* **(e)**, induced by rh-TRAIL (10 ng/ml), is inhibited by a JNK inhibitor, SP-600125 (3–30 μM). **(c)** Mmp-9 and Timp-1 protein are induced by rh-TRAIL (10 ng/ml) after 2 and 0.5 hours, respectively. Samples were in triplicate (or more) for each group. Data are presented as mean ± SD; *p<0.05 or **p<0.01, as compared to controls or rh-TRAIL alone.
(EPS)

S8 Fig A Model for Development of AAA With or Without *Opg.* Schematic diagram of AAA formation in the abdominal aorta in wild-type and *Opg-*KO mice. **(a)** CaCl_2_ application is schematically shown. **(b)** In the acute phase, dilation of internal aortic dimensions, thinning of the medial layer (red arrows), and thickening of the adventitia occur. **(c)** In the late phase, vascular size appears to be recovered, but linear elastic lamella structure remains within the media and inflammatory cells are found in the adventitia of wild-type mice. In contrast, increased vascular size and thickening of both the media and adventitia is observed continuously in *Opg-*KO mice. EC: endothelial cells; SMC: smooth muscle cells.
(EPS)

## References

[1] Ailawadi G, Eliason JL, Upchurch GR Jr. (2003) Current concepts in the pathogenesis of abdominal aortic aneurysm. J Vasc Surg 38: 584–588. 12947280 10.1016/s0741-5214(03)00324-0

[2] Golledge J, Norman PE (2010) Atherosclerosis and abdominal aortic aneurysm: cause, response, or common risk factors? Arterioscler Thromb Vasc Biol 30: 1075–1077. 10.1161/ATVBAHA.110.206573 20484703 PMC2874982

[3] Golledge J, Muller J, Daugherty A, Norman P (2006) Abdominal aortic aneurysm: pathogenesis and implications for management. Arterioscler Thromb Vasc Biol 26: 2605–2613. 16973970 10.1161/01.ATV.0000245819.32762.cb

[4] Pearce WH, Koch AE (1996) Cellular components and features of immune response in abdominal aortic aneurysms. Ann N Y Acad Sci 800: 175–185. 8958992 10.1111/j.1749-6632.1996.tb33308.x

[5] Bobryshev YV, Lord RS, Parsson H (1998) Immunophenotypic analysis of the aortic aneurysm wall suggests that vascular dendritic cells are involved in immune responses. Cardiovasc Surg 6: 240–249. 9705095 10.1177/096721099800600305

[6] Freestone T, Turner RJ, Coady A, Higman DJ, Greenhalgh RM, et al (1995) Inflammation and matrix metalloproteinases in the enlarging abdominal aortic aneurysm. Arterioscler Thromb Vasc Biol 15: 1145–1151. 7627708 10.1161/01.atv.15.8.1145

[7] Thompson EW, Blackshaw AW, Raychoudhury SS (1995) Secreted products and extracellular matrix from testicular peritubular myoid cells influence androgen-binding protein secretion by Sertoli cells in culture. J Androl 16: 28–35. 7768750

[8] Davis V, Persidskaia R, Baca-Regen L, Itoh Y, Nagase H, et al (1998) Matrix metalloproteinase-2 production and its binding to the matrix are increased in abdominal aortic aneurysms. Arterioscler Thromb Vasc Biol 18: 1625–1633. 9763536 10.1161/01.atv.18.10.1625

[9] Longo GM, Xiong W, Greiner TC, Zhao Y, Fiotti N, et al (2002) Matrix metalloproteinases 2 and 9 work in concert to produce aortic aneurysms. J Clin Invest 110: 625–632. 12208863 10.1172/JCI15334 PMC151106

[10] Yamashita A, Noma T, Nakazawa A, Saito S, Fujioka K, et al (2001) Enhanced expression of matrix metalloproteinase-9 in abdominal aortic aneurysms. World J Surg 25: 259–265. 11343173 10.1007/s002680020062

[11] Elmore JR, Keister BF, Franklin DP, Youkey JR, Carey DJ (1998) Expression of matrix metalloproteinases and TIMPs in human abdominal aortic aneurysms. Ann Vasc Surg 12: 221–228. 9588507 10.1007/s100169900144

[12] Eskandari MK, Vijungco JD, Flores A, Borensztajn J, Shively V, et al (2005) Enhanced abdominal aortic aneurysm in TIMP-1-deficient mice. J Surg Res 123: 289–293. 15680392 10.1016/j.jss.2004.07.247

[13] Yoshimura K, Aoki H, Ikeda Y, Fujii K, Akiyama N, et al (2005) Regression of abdominal aortic aneurysm by inhibition of c-Jun N-terminal kinase. Nat Med 11: 1330–1338. 16311603 10.1038/nm1335

[14] Baud'huin M, Duplomb L, Teletchea S, Lamoureux F, Ruiz-Velasco C, et al (2013) Osteoprotegerin: multiple partners for multiple functions. Cytokine Growth Factor Rev 24: 401–409. 10.1016/j.cytogfr.2013.06.001 23827649

[15] Simonet WS, Lacey DL, Dunstan CR, Kelley M, Chang MS, et al (1997) Osteoprotegerin: a novel secreted protein involved in the regulation of bone density. Cell 89: 309–319. 9108485 10.1016/s0092-8674(00)80209-3

[16] Sims NA, Gooi JH (2008) Bone remodeling: Multiple cellular interactions required for coupling of bone formation and resorption. Semin Cell Dev Biol 19: 444–451. 10.1016/j.semcdb.2008.07.016 18718546

[17] Kimberley FC, Screaton GR (2004) Following a TRAIL: update on a ligand and its five receptors. Cell Res 14: 359–372. 15538968 10.1038/sj.cr.7290236

[18] Bucay N, Sarosi I, Dunstan CR, Morony S, Tarpley J, et al (1998) osteoprotegerin-deficient mice develop early onset osteoporosis and arterial calcification. Genes Dev 12: 1260–1268. 9573043 10.1101/gad.12.9.1260 PMC316769

[19] Price PA, June HH, Buckley JR, Williamson MK (2001) Osteoprotegerin inhibits artery calcification induced by warfarin and by vitamin D. Arterioscler Thromb Vasc Biol 21: 1610–1616. 11597934 10.1161/hq1001.097102

[20] Bennett BJ, Scatena M, Kirk EA, Rattazzi M, Varon RM, et al (2006) Osteoprotegerin inactivation accelerates advanced atherosclerotic lesion progression and calcification in older ApoE-/- mice. Arterioscler Thromb Vasc Biol 26: 2117–2124. 16840715 10.1161/01.ATV.0000236428.91125.e6

[21] Orita Y, Yamamoto H, Kohno N, Sugihara M, Honda H, et al (2007) Role of osteoprotegerin in arterial calcification: development of new animal model. Arterioscler Thromb Vasc Biol 27: 2058–2064. 17615383 10.1161/ATVBAHA.107.147868

[22] Morony S, Tintut Y, Zhang Z, Cattley RC, Van G, et al (2008) Osteoprotegerin inhibits vascular calcification without affecting atherosclerosis in ldlr(-/-) mice. Circulation 117: 411–420. 10.1161/CIRCULATIONAHA.107.707380 18172035 PMC2680735

[23] Collin-Osdoby P (2004) Regulation of vascular calcification by osteoclast regulatory factors RANKL and osteoprotegerin. Circ Res 95: 1046–1057. 15564564 10.1161/01.RES.0000149165.99974.12

[24] Moran CS, Jose RJ, Biros E, Golledge J (2014) Osteoprotegerin Deficiency Limits Angiotensin II-Induced Aortic Dilatation and Rupture in the Apolipoprotein E-Knockout Mouse. Arterioscler Thromb Vasc Biol 34: 2609–2616. 10.1161/ATVBAHA.114.304587 25301844 PMC4239170

[25] Jono S, Ikari Y, Shioi A, Mori K, Miki T, et al (2002) Serum osteoprotegerin levels are associated with the presence and severity of coronary artery disease. Circulation 106: 1192–1194. 12208791 10.1161/01.cir.0000031524.49139.29

[26] Schoppet M, Sattler AM, Schaefer JR, Herzum M, Maisch B, et al (2003) Increased osteoprotegerin serum levels in men with coronary artery disease. J Clin Endocrinol Metab 88: 1024–1028. 12629080 10.1210/jc.2002-020775

[27] Moran CS, McCann M, Karan M, Norman P, Ketheesan N, et al (2005) Association of osteoprotegerin with human abdominal aortic aneurysm progression. Circulation 111: 3119–3125. 15939823 10.1161/CIRCULATIONAHA.104.464727

[28] Koole D, Hurks R, Schoneveld A, Vink A, Golledge J, et al (2012) Osteoprotegerin is associated with aneurysm diameter and proteolysis in abdominal aortic aneurysm disease. Arterioscler Thromb Vasc Biol 32: 1497–1504. 10.1161/ATVBAHA.111.243592 22516062

[29] Travo P, Barrett G, Burnstock G (1980) Differences in proliferation of primary cultures of vascular smooth muscle cells taken from male and female rats. Blood Vessels 17: 110–116. 7362876 10.1159/000158240

[30] Zhang X, Goncalves R, Mosser DM (2008) The isolation and characterization of murine macrophages. Curr Protoc Immunol Chapter 14: Unit 14 11. 10.1002/0471142735.im1401s83 PMC2834554 19016445

[31] Wei W, Wang D, Shi J, Xiang Y, Zhang Y, et al Tumor necrosis factor (TNF)-related apoptosis-inducing ligand (TRAIL) induces chemotactic migration of monocytes via a death receptor 4-mediated RhoGTPase pathway. Mol Immunol 47: 2475–2484. 10.1016/j.molimm.2010.06.004 20638129

[32] Soe NN, Ishida T, Ishida M, Sawano M, Abe K, et al (2009) Nifedipine interferes with migration of vascular smooth muscle cells via inhibition of Pyk2-Src axis. J Atheroscler Thromb 16: 230–238. 19556728 10.5551/jat.e422

[33] Olesen P, Ledet T, Rasmussen LM (2005) Arterial osteoprotegerin: increased amounts in diabetes and modifiable synthesis from vascular smooth muscle cells by insulin and TNF-alpha. Diabetologia 48: 561–568. 15700136 10.1007/s00125-004-1652-8

[34] Kavurma MM, Schoppet M, Bobryshev YV, Khachigian LM, Bennett MR (2008) TRAIL stimulates proliferation of vascular smooth muscle cells via activation of NF-kappaB and induction of insulin-like growth factor-1 receptor. J Biol Chem 283: 7754–7762. 10.1074/jbc.M706927200 18178561

[35] Bumdelger B, Kokubo H, Kamata R, Fujii M, Ishida M, et al (2013) Induction of Timp1 in smooth muscle cells during development of abdominal aortic aneurysms. Hiroshima J Med Sci 62: 63–67. 24279124

[36] Papalambros E, Sigala F, Georgopoulos S, Menekakos C, Giatromanolaki A, et al (2003) Immunohistochemical expression of metalloproteinases MMP-2 and MMP-9 in abdominal aortic aneurysms: correlation with symptoms and aortic diameter. Int J Mol Med 12: 965–968. 14612975

[37] Newby AC (2005) Dual role of matrix metalloproteinases (matrixins) in intimal thickening and atherosclerotic plaque rupture. Physiol Rev 85: 1–31. 15618476 10.1152/physrev.00048.2003

[38] Folkesson M, Kazi M, Zhu C, Silveira A, Hemdahl AL, et al (2007) Presence of NGAL/MMP-9 complexes in human abdominal aortic aneurysms. Thromb Haemost 98: 427–433. 17721627

[39] Muhlenbeck F, Haas E, Schwenzer R, Schubert G, Grell M, et al (1998) TRAIL/Apo2L activates c-Jun NH2-terminal kinase (JNK) via caspase-dependent and caspase-independent pathways. J Biol Chem 273: 33091–33098. 9830064 10.1074/jbc.273.49.33091

[40] Hu WH, Johnson H, Shu HB (1999) Tumor necrosis factor-related apoptosis-inducing ligand receptors signal NF-kappaB and JNK activation and apoptosis through distinct pathways. J Biol Chem 274: 30603–30610. 10521444 10.1074/jbc.274.43.30603

[41] Forte A, Della Corte A, De Feo M, Cerasuolo F, Cipollaro M (2010) Role of myofibroblasts in vascular remodelling: focus on restenosis and aneurysm. Cardiovasc Res 88: 395–405. 10.1093/cvr/cvq224 20621923

[42] Ovchinnikova O, Gylfe A, Bailey L, Nordstrom A, Rudling M, et al (2009) Osteoprotegerin promotes fibrous cap formation in atherosclerotic lesions of ApoE-deficient mice—brief report. Arterioscler Thromb Vasc Biol 29: 1478–1480. 10.1161/ATVBAHA.109.188185 19592469

[43] Uzui H, Morishita T, Nakano A, Amaya N, Fukuoka Y, et al (2014) Effects of combination therapy with olmesartan and azelnidipine on serum osteoprotegerin in patients with hypertension. J Cardiovasc Pharmacol Ther 19: 304–309. 10.1177/1074248413511692 24288395

[44] Jono S, Otsuki S, Higashikuni Y, Shioi A, Mori K, et al (2010) Serum osteoprotegerin levels and long-term prognosis in subjects with stable coronary artery disease. J Thromb Haemost 8: 1170–1175. 10.1111/j.1538-7836.2010.03833.x 20230427

[45] Raffetto JD, Khalil RA (2008) Matrix metalloproteinases and their inhibitors in vascular remodeling and vascular disease. Biochem Pharmacol 75: 346–359. 17678629 10.1016/j.bcp.2007.07.004 PMC2254136

[46] Maiellaro K, Taylor WR (2007) The role of the adventitia in vascular inflammation. Cardiovasc Res 75: 640–648. 17662969 10.1016/j.cardiores.2007.06.023 PMC3263364

